# Interprofessional team interactions about complex care in the ICU: pilot development of an observational rating tool

**DOI:** 10.1186/s13104-016-2213-1

**Published:** 2016-08-18

**Authors:** Deena Kelly Costa, Jennifer Dammeyer, Matthew White, Jose Galinato, Robert Hyzy, Milisa Manojlovich, Anne Sales

**Affiliations:** 1 Department of Systems, Populations & Leadership, School of Nursing, University of Michigan, 400 North Ingalls 4351, Ann Arbor, MI USA; 2 University of Michigan Hospital System, Ann Arbor, MI USA; 3 Division of Pulmonary and Critical Care Medicine, School of Medicine, University of Michigan, Ann Arbor, MI USA; 4 Trinity Health System, Victor Parkway, Livonia, MI USA; 5 Department of Learning Health Systems, School of Medicine, University of Michigan, Ann Arbor, MI USA; 6 Center for Clinical Management Research, VA Ann Arbor Healthcare System, Ann Arbor, MI USA

**Keywords:** Critical care, Teamwork, Complex care delivery, Mechanical ventilation

## Abstract

**Background:**

The awakening and breathing coordination, delirium, and early mobility (ABCDE) bundle is a multicomponent complex intervention that improves outcomes for critically ill adults yet is inconsistently implemented. Effective interprofessional team function (how the team interacts) is key to ABCDE delivery but little is known about how to measure team interactions. The purpose of our study was to examine the reliability of an observational rating tool to assess team interactions about ABCDE in one ICU.

**Results:**

We pilot tested and evaluated reliability of an observational rating tool to assess team interactions about ABCDE. Two independent raters used this tool in one medical ICU over 4 weeks during morning rounds. We examined which ABCDE components were addressed, which team members initiated interactions, and which participated in interactions about ABCDE. We evaluated inter-rater reliability using Cohen’s kappa statistic and data from interprofessional team interactions for 23 patients. We demonstrated moderate to substantial reliability for whether breathing, coordination, delirium or early mobility were addressed (k = 0.48–0.78) and slight to fair reliability for which team members initiated interactions about ABCDE (0.18–0.40). Reliability was low for whether Awakening was addressed (k = −0.07) and for which team members initiated interactions about awakening (k = 0.05).

**Conclusions:**

Our study provides pilot evidence of reliability of an observational rating tool to assess interprofessional team interactions about ABCDE. Future work should further test and modify this tool to gain an understanding of how to use team interactions to improve ABCDE delivery.

**Electronic supplementary material:**

The online version of this article (doi:10.1186/s13104-016-2213-1) contains supplementary material, which is available to authorized users.

## Background

Increasingly intensive care unit (ICU) clinicians are aware of the long-term, detrimental effects of ICU care [1]. Limited mobility and lengthy duration of mechanical ventilation can contribute to long-term functional and cognitive decline [1, 2]. The Awakening and Breathing, Coordination, Delirium monitoring/management, and Early exercise/mobility bundle (ABCDE) is a multicomponent evidence-based care bundle shown to reduce duration of mechanical ventilation, lower odds of delirium, and improve physical mobility for critically ill adults [3–5]. Despite the safety and effectiveness of the ABCDE bundle, prior studies have found that ABCDE is difficult to integrate into every day practice and remains inconsistently delivered [6].

The interprofessional team plays a unique role in ABCDE delivery. Effective interprofessional team function (i.e. how the team interacts to deliver care) is cited as key to successful implementation [7, 8] but is difficult to assess. Barriers to examining team function, specifically interactions about ABCDE, may be due to difficulties with measuring interactions in the clinical setting. Morning rounds, where the team comes together to discuss plans of care, may offer an opportunity to assess team interactions and identify potential targets for future quality improvement projects to facilitate ABCDE delivery. Thus, we pilot tested an observation tool used during morning rounds to rate: (1) what components of the ABCDE bundle were addressed (2) which interprofessional team members initiated interactions and (3) which interprofessional team members participated in interactions. We sought to determine the reliability of this observational tool to assess ICU interprofessional team interactions to offer insight into how teams function to deliver evidence based care.

## Methods

The University of Michigan Institutional Review Board reviewed this study and granted a determination of ‘not regulated’ status (HUM00104862).

### Setting and sample

We pilot-tested the observational tool during morning rounds in one medical ICU in an academic medical center. In this 20-bed medical ICU, rounds occur at least once daily and are led by the attending physician or critical care fellow. There is a structured format to rounds, which includes the use of a daily goals sheet (see Additional file 1). At the doorway of each patient room, the intern or resident assigned to that patient presents the case, focusing on overnight events, current status, and plans for the day. The attending is encouraged to incorporate the bedside nurse in rounds, which may include asking the nurse about ABCDE, but nurses are not consistently present on rounds. Other disciplines present during rounds may include: respiratory therapists, clinical pharmacists, and registered dieticians.

Two independent raters observed all clinician interactions during morning rounds, 1 day a week, selected at random, for 1 month (November 20, 2014–December 20, 2014). We observed team interactions for all patients, regardless of mechanical ventilation status, because clinicians in this ICU were encouraged to discuss ABCDE on all patients via their daily goals sheet. In our observations, we attempted to minimize any Hawthorne effect by ensuring that even though the ICU team was aware of our presence, they did not know we were evaluating interactions about ABCDE.

### Design

To develop our semi-structured observational rating tool, we adapted a tool from a prospective trial [3], conducted a review of the literature, and sought input from clinicians. We created categories for each individual component of ABCDE with domains for: (1) what components of ABCDE were addressed (2) which interprofessional team members initiated interactions about ABCDE; and (3) what other interprofessional team members participated in these interactions. These three components of interactions about ABCDE were important to measure as they offer insight into whether certain bundle components are discussed more or less frequently and by certain providers; providing guidance about targets for future quality improvement work. We reviewed the literature to derive the operational definitions of the tool components and combined what we found with the operational definitions from an enrollment tool used in a prospective trial [3]. We defined which interprofessional team member initiated interactions as the interprofessional team member who first mentioned ABCDE. For example, if the ICU nurse first mentioned ABCDE in his/her report, then the ICU nurse was categorized as initiating the interaction. We defined which interprofessional team members participated in interactions about ABCDE as any interprofessional team member who added information, after the initial interaction about ABCDE.

We assessed content validity by obtaining feedback from five different clinicians (3 critical care nurses, one acute care nurse, and one nurse researcher) before trialing and piloting the tool. We (DKC and JG) trialed the tool in the ICU for 2 days and modified it to include more detail about potential items for discussion for each component. Then, we (DKC & JG) observed morning rounds but independently rated team interactions.

## Analysis

We analyzed only team interactions where both raters were present. We tested the reliability of the observational rating tool by evaluating inter-rater reliability using Cohen’s kappa statistic, a well-known standard for the strength of agreement between raters [9].

## Results

Both raters individually rated team interactions for 23 unique patients within one medical ICU during a 4-week period.

**Fig. 1:**
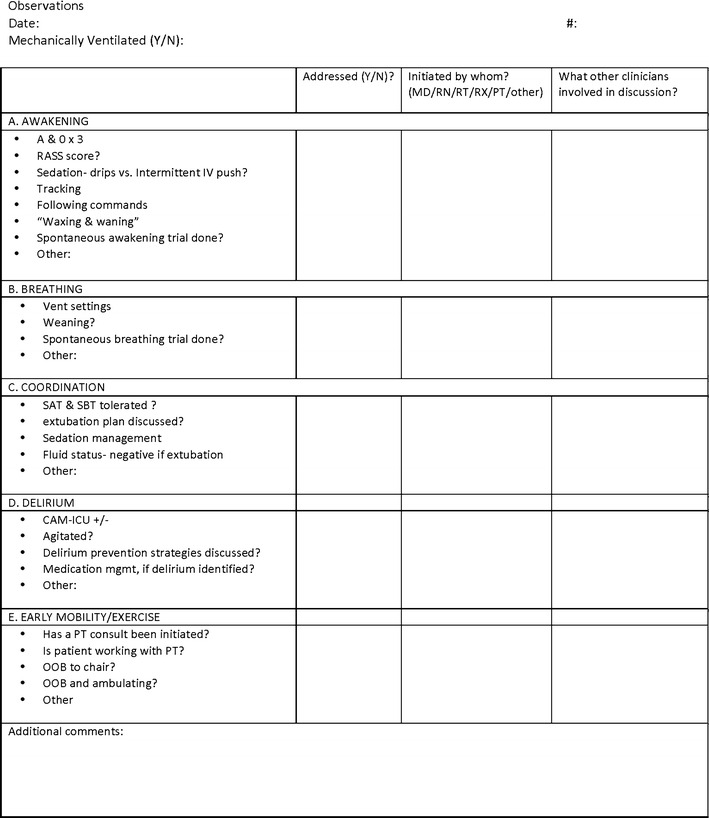
ABCDE interactions observational rating tool

The observational rating tool is displayed in Fig. 1.

Table 1 displays the results of the inter-rater reliability of our observational rating tool including percent agreement between raters and Cohen’s kappa coefficients with corresponding 95 % confidence intervals. Percent agreement between raters for whether individual bundle components were addressed was high, ranging from 76 to 91 % but was lower for which clinicians initiated interactions (30–69 %) (see Table 1). We demonstrated moderate to substantial inter-rater reliability for whether ‘Breathing’, ‘Coordination’, ‘Delirium’, or ‘Early mobility’ was addressed during team interactions (k = 0.48–0.78) [9]. However, inter-rater reliability was poor (k = −0.07) for whether ‘Awakening’ was addressed. Inter-rater reliability for which interprofessional team members initiated discussions was slight to fair for, ‘Breathing’, ‘Coordination’, ‘Delirium’ and ‘Early mobility’ (kappa = 0.18–0.40) but lower for ‘Awakening’ (k = 0.05). We were unable to evaluate inter-rater reliability for the item that assessed which other clinicians participated in these interactions since there were too few ratings in our data.

**Table 1:** Percent agreement and Cohen’s kappa coefficients for each component of the observational rating tool by two raters (n = 23 patient care team interactions)

	Addressed		Initiated	
	% Agreement	Kappa (95 % CI)	% Agreement	Kappa (95 % CI)
A: Awakening	78	k = −0.07 (−0.22,−0.07)	30	k = 0.05 (0.05, 0.32)
B: Breathing	91	k = 0.62 (0.16, 1.0)	50	k = 0.27 (−0.01, 0.35)
C: Coordination	79	k = 0.55 (0.18, 0.93)	62	k = 0.40 (0.25, 0.47)
D: Delirium	76	k = 0.48 (0.01, 0.87)	69	k = 0.18 (−0.19, 0.34)
E: Early mobility	89	k = 0.78 (0.50, 1.0)	61	k = 0.39 (0.24, 0.60)

The third column of the observational rating tool, “what other clinicians participated in these conversations?” had too few ratings to reliably assess agreement and inter-rater reliability and is thus excluded from Table 1

*CI* confidence interval

## Conclusions

This study provides pilot evidence of the reliability of an observational rating tool to assess interprofessional team interactions about ABCDE in one ICU.

We demonstrated the highest inter-rater reliability for which components of the bundle were addressed during team interactions and most specifically, for ‘Breathing’, ‘Coordination’, ‘Delirium,’ and ‘Early mobility’ components (Table 1). Yet, reliability for the item that rated whether ‘Awakening’ was addressed (k = −0.07) was much lower. There are a couple of reasons for our finding of lower reliability for whether ‘Awakening’ was addressed. First, it is possible that during morning rounds in a large and busy ICU, raters may not have uniformly heard the interaction about ‘Awakening’ when transitioning from one patient to another. Second, ‘Awakening’ may not have been addressed at all, which would potentially explain the lower reliability of this component of the observational rating tool.

Low inter-rater reliability for one component of the bundle also highlights the complexity of assessing team interactions in the clinical setting. Two raters were present and observed the same team interactions but interactions occur in a fluid, dynamic context. Rounds in the USA can be large with representation from at least four professions—a physician, a nurse, a pharmacist and a respiratory therapist [10]—in addition to any trainees in an academic setting. Since the ABCDE bundle is not yet a routinized part of care [6] and discussion of ‘Awakening’ may be first, we would expect variability in rating team interactions about ‘Awakening’ in a large ICU with multiple clinicians present during morning rounds. Further, not all team members may actively engage or participate in interactions about ABCDE even when present which could influence measurement. Recent data suggest that some clinicians do not actively engage in morning rounds despite having pertinent knowledge [11]. Lack of engagement and participation of ICU teams may also have an adverse effect on potential quality improvement projects. Indeed, lack of engagement by clinicians to ABCDE delivery is cited as a frequent barrier [7].

We demonstrated slight to fair reliability for the items about initiation of interactions about ABCDE but were unable to assess inter-rater reliability for participation in interactions about ABCDE. We suspect that it was difficult to reliably classify interprofessional team member participation using the tool because of the free-form documentation format. Each rater could identify the team member who participated, in his or her own words, which may not be equivalent. Free-form text may not be the most appropriate way to assess participation and future modifications to the tool should include defined options for this domain (i.e. check boxes for each clinician type). Further, no option was available on the observational rating tool to indicate that no other clinician participated in the interaction (besides those that initiated the interaction). This may have contributed to too few ratings on the participation domain and thus our inability to evaluate reliability. Given these findings and the complexity in documenting the number and type of interprofessional team members, we intend to modify the tool for future work to include check boxes to select team member participation.

Despite being one of the first studies to develop an observational rating tool to assess team interactions about ABCDE, our study does have limitations. First, this study was conducted in one ICU in a large academic medical center in the Midwest and our results may not be generalizable. Second, we present pilot data of the development of an observational rating tool and we are limited by a small sample size and potential lack of power. As such, we focus in this article on the psychometrics properties and tool development. Lastly, our operational definitions of the individual bundle components, although informed by a review of a literature and an enrollment tool from a prospective ABCDE trial, may require further clarification and we acknowledge this limitation.

In conclusion, we find moderate to substantial reliability of an observational rating tool to assess team interactions about ABCDE in one medical ICU. We find slight to fair reliability when assessing which team members initiated interactions about ABCDE but were unable to assess team member participation in interactions about ABCDE. Future work should focus on further testing of this tool to understand how information about team interactions could be leveraged to improve delivery of a complex care bundle like ABCDE.

## Additional file

10.1186/s13104-016-2213-1 ICU daily goals sheet.

## Abbreviations

ICU: intensive care unit
ABCDE: awakening and breathing coordination, delirium monitoring/management, and early exercise/mobility (ABCDE) bundle

## References

[1] Vasilevskis EE, Ely EW, Speroff T, Pun BT, Boehm L, Dittus RS (2010). Reducing iatrogenic risks: ICU-acquired delirium and weakness—crossing the quality chasm Chest. Am Coll Chest Phys.

[2] Herridge MS, Chu L, Matte A, Tomlinson G, Chan L, Thomas C (2015). The RECOVER program: one-year disability in critically ill patients mechanically ventilated (MV) for 7 days. Am J Respir Crit Care Med.

[3] Balas MC, Vasilevskis EE, Olsen KM, Schmid KK, Shostrom V, Cohen MZ (2014). Effectiveness and safety of the awakening and breathing coordination, delirium monitoring/management, and early exercise/mobility bundle. Crit Care Med.

[4] Girard TD, Kress JP, Fuchs BD, Thomason JW, Schweickert WD, Pun BT (2008). Efficacy and safety of a paired sedation and ventilator weaning protocol for mechanically ventilated patients in intensive care (awakening and breathing controlled trial): a randomised controlled trial. Lancet.

[5] Khan B, Fadel WF, Tricker JL, Carlos WG, Farber MO, Hui SL (2014). Effectiveness of implementing a wake up and breathe program on sedation and delirium in the ICU. Crit Care Med.

[6] Miller MA, Govindan S, Watson SR, Hyzy RC, Iwashyna TJ (2015). ABCDE, but in that order? A cross-sectional survey of michigan icu sedation, delirium and early mobility practices. Ann Am Thorac Soc.

[7] Carrothers KM, Barr J, Spurlock B, Ridgely MS, Damberg CL, Ely EW (2013). Contextual issues influencing implementation and outcomes associated with an integrated approach to managing pain, agitation, and delirium in adult ICUs. Crit Care Med.

[8] Balas MC, Burke WJ, Gannon D, Cohen MZ, Colburn L, Bevil C (2013). Implementing the awakening and breathing coordination, delirium monitoring/management, and early exercise/mobility bundle into everyday care: opportunities, challenges, and lessons learned for implementing the ICU pain, agitation, and delirium guidelines. Crit Care Med.

[9] Landis JR, Koch GG (1977). The measurement of observer agreement for categorical data. Biometrics.

[10] Kim MM, Barnato AE, Angus DC, Fleisher LA, Kahn JM (2010). The effect of multidisciplinary care teams on intensive care unit mortality. Arch Intern Med.

[11] Alexanian JA, Kitto S, Rak KJ, Reeves S (2015). Beyond the team. Crit Care Med.

