# Supplementary figures and images for: Interprofessional team interactions about complex care in the ICU: pilot development of an observational rating tool
Source: BMC Res Notes. 2016 Aug 18;9:408. doi: 10.1186/s13104-016-2213-1 (PMC4990869; doi:10.1186/s13104-016-2213-1)

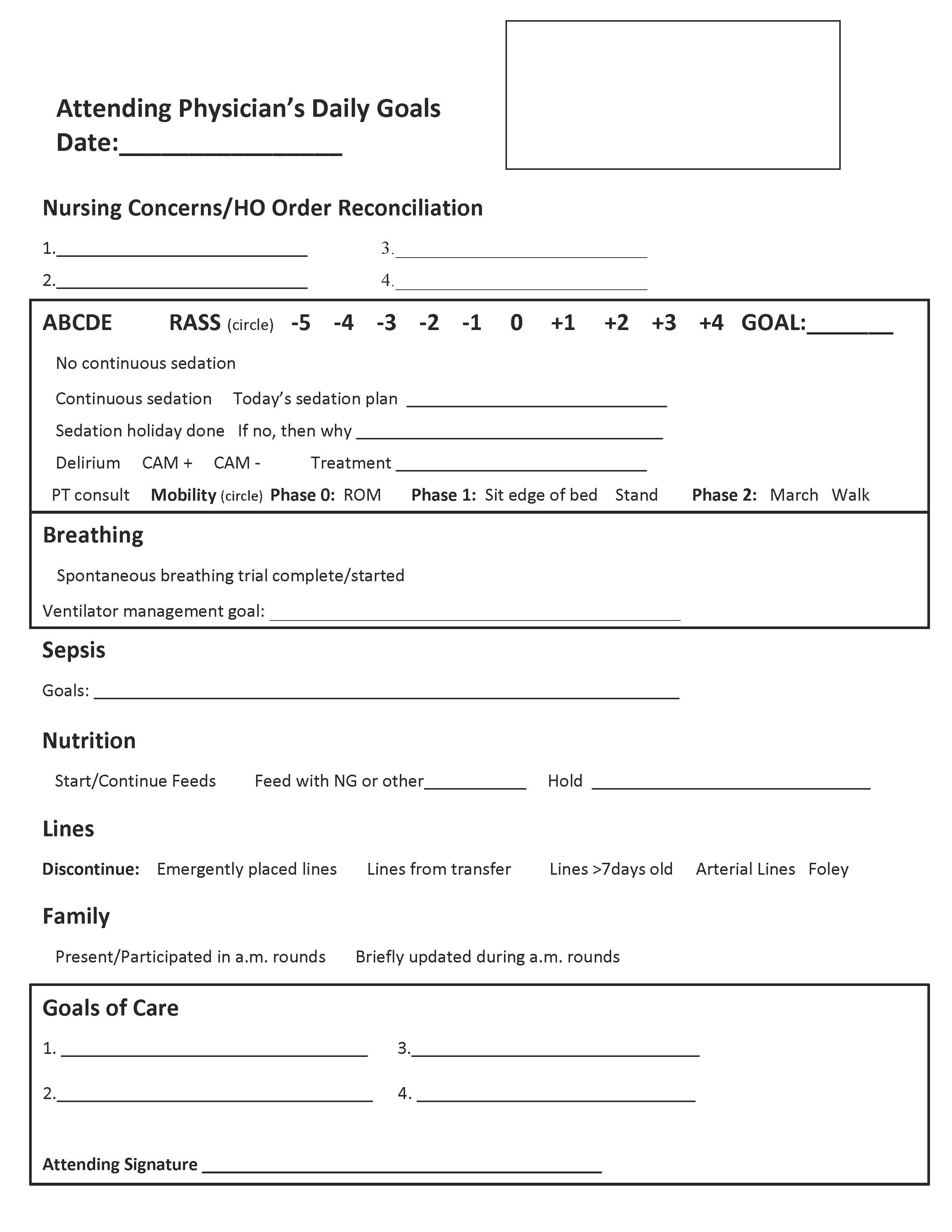

Supplement: Supplementary file 1 — 10.1186/s13104-016-2213-1 ICU daily goals sheet. [file 13104_2016_2213_MOESM1_ESM.jpg]
